# Prognostic value of ERBB4 expression in patients with triple negative breast cancer

**DOI:** 10.1186/s12885-016-2195-3

**Published:** 2016-02-22

**Authors:** Ji-Yeon Kim, Hae Hyun Jung, In-Gu Do, SooYoun Bae, Se Kyung Lee, Seok Won Kim, Jeong Eon Lee, Seok Jin Nam, Jin Seok Ahn, Yeon Hee Park, Young-Hyuck Im

**Affiliations:** Division of Hematology-Oncology, Department of Medicine, Samsung Medical Center, Sungkyunkwan University School of Medicine, 81 Irwon-ro, Gangnam-gu, Seoul, 135-710 Korea; Biomedical Research Institute, Samsung Medical Center, Sungkyunkwan University School of Medicine, 81 Irwon-ro, Gangnam-gu, Seoul, 135-710 Korea; Cancer of Companion Diagnostics, Innovative Cancer Medicine Institute, Samsung Medical Center, Sungkyunkwan University School of Medicine, 81 Irwon-ro, Gangnam-gu, Seoul, 135-710 Korea; Department of Surgery, Samsung Medical Center, Sungkyunkwan University School of Medicine, 81 Irwon-ro, Gangnam-gu, Seoul, 135-710 Korea

**Keywords:** ERBB4, Triple negative breast cancer, nCounter expression assay

## Abstract

**Background:**

Triple-negative breast cancer (TNBC) is known for aggressive biologic features and poor prognosis. Epidermal growth factor receptor (EGFR) overexpression in TNBC indicates poor prognosis. However, there is no previous study of the relationship between expression of the entire human epidermal growth factor receptor (HER) family genes and patient prognosis in TNBC. Accordingly, we investigated the expression profiles of HER family genes in patients with TNBC to determine the prognostic value and clinical implications of HER family expression.

**Methods:**

We used the nCounter expression assay (NanoString®) to measure the expression of EGFR, erb-B2 receptor tyrosine kinase 2 (ERBB2), ERBB3, ERBB4, and estrogen receptor 1 (ESR1) genes using mRNA extracted from paraffin-embedded tumor tissues from 203 patients diagnosed with TNBC. Our data were validated using a separate cohort of 84 TNBC patients.

**Results:**

A total of 203 TNBC patients who received adjuvant chemotherapy after curative surgery from 2000 to 2004 formed the training set. The 84 TNBC patients in the validation consort were selected from breast cancer patients who received curative surgery since 2005 to 2010. Analysis of the expression profiles of the HER family genes in TNBC tissue specimens revealed that increased expression of ERBB4 was associated with poor prognosis according to survival analysis (5-year distant relapse free survival [5Y DRFS], low vs. high expression [cut-off: median]: 90.1 % vs. 80.2 %; *p* = 0.022). This trend was also observed in the validation set of TNBC patients (5Y DRFS, low vs. high: 69.4 % vs. 44.7 %; *p* = 0.053). In a multivariate Cox regression model, ERBB4 expression was identified as a indicator of long-term prognosis in patients with TNBC.

**Conclusions:**

The expression profile of ERBB4, a member of the HER family, might serve as a prognostic marker in patients with TNBC.

**Electronic supplementary material:**

The online version of this article (doi:10.1186/s12885-016-2195-3) contains supplementary material, which is available to authorized users.

## Background

Triple negative breast cancer (TNBC), defined as the absence of both hormone receptor expression and erb-B2 receptor tyrosine kinase 2 (ERBB2) overexpression, accounts for approximately 15-20 % of all breast cancers [1]. In general, TNBC is diagnosed at a higher stage and has more aggressive biologic features and worse prognosis than other subtypes [2, 3].

Overexpression of the human epidermal growth factor receptor (HER) family members, consisting of epidermal growth factor receptor (EGFR), ERBB2, ERBB3, and ERBB4, is frequently observed in many kinds of human epithelial malignancies [4]. Of the four HER family members, ERBB2 overexpression is known to induce carcinogenesis in mammalian cells [5, 6]. ERBB2 overexpression is found in 15-20 % of breast cancers and defines a unique subtype of breast cancer [7]. Indeed, ERBB2 overexpression is the therapeutic target for the monoclonal antibodies trastuzumab and pertuzumab and the tyrosine kinase inhibitor lapatinib [8–11].

In addition to the known role of ERBB2, research on other HER family genes in breast cancer is now ongoing. According to previous research, overexpression of EGFR, ERBB2, and ERBB3 is associated with poor prognosis and negatively correlated with estrogen receptor (ER) expression in breast cancer [12, 13].

In terms of ERBB4, intracellular domain 4ICD of ERBB4 promotes apoptosis of breast cancer cells and cytosolic expression of 4ICD is associated with good prognosis [14, 15]. Moreover, ERBB4 expression is significantly related to levels of phospho-AKT and ERK in TNBC as a good prognostic factor [16]. Another study reported that ERBB4 expression is positively related to ER-positive breast cancer [17–19]. Patients with breast cancer showing co-expression of ERBB4 and ER have fewer recurrences and improved survival compared to patients diagnosed with ER-positive breast cancer without ERBB4 expression [20, 21].

Some studies have found that EGFR overexpression indicates poor prognosis in TNBC [22, 23]. In preclinical studies, EGFR overexpression was detected more frequently and at higher levels in TNBC cell lines than in other subtypes and the combination of an EGFR targeting agent and cytotoxic agent inhibited cell growth more effectively than cytotoxic chemotherapy alone [24]. The results of phase I/II clinical trials of cetuximab, a monoclonal antibody targeting EGFR overexpression, demonstrated clinical benefit in TNBC with EGFR overexpression [25–27]. However, there are no clear data supporting the clinical significance of expression of the entire HER family genes in TNBC.

Accordingly, we determined how the mRNA expression levels of HER family genes affect the prognosis of patients with TNBC.

## Methods

### Patients

This study was a retrospective analysis of the clinical records of patients with invasive breast cancer who received adjuvant chemotherapy after curative surgery at Samsung Medical Center between 2000 and 2004. Women who were diagnosed with breast cancer at stage I to IIIC by diagnostic examination (breast magnetic resonance imaging [MRI], abdominal computed tomography [CT] scan, bone scan, and/or positron emission tomography [PET]-CT scans if indicated) were included in the training cohort. To validate our data, we retrospectively reviewed clinical records of breast cancer patients who received curative surgery at Samsung Medical Center from 2005 to 2010.

The institutional review board of Samsung Medical Center, Seoul, Korea approved our study protocol and waived the need for informed consent due to this study was conducted using archival tissues with retrospective clinical data (IRB No: 2012-08-065).

### Immunohistochemistry and RNA extraction

We obtained all available hematoxylin and eosin (H&E)-stained slides of archival formalin-fixed, paraffin-embedded (FFPE) primary breast tumor tissue samples. Two independent pathologists reviewed all pathology specimens to determine tumor histologic characteristics (histological grade [28] and nuclear grade) and immunohistochemical (IHC) findings (ER and progesterone receptor [PgR] expression and ERBB2 overexpression). ER and PgR positivity were defined using Allred scores ranging from 3 to 8 based on IHC using antibodies to ER (Immunotech, Marseille, France) and PgR (Novocastra Laboratories Ltd., Newcastle upon Tyne, UK). HER2 status was evaluated using a specific antibody (Dako, Glostrop, Denmark) and/or silver in situ hybridization (SISH). Grades 0 and 1 for ERBB2, as assessed by IHC, were defined as a negative result, and grade 3 was defined as a positive result. Amplification of ERBB2 was confirmed by SISH if ERBB2 was rated as 2+ by IHC. TNBC was defined as a negative result for ER/PgR and ERBB2.

RNA was extracted from 2–4 sections of 4-μm thick FFPE sections containing more than 75 percent of tumor cells in tumor tissue using the High Pure RNA Paraffin kit (Roche Diagnostic, Mannheim, Germany). RNA yield and purity were assessed using a NanoDrop ND-1000 Spectrophotometer (NanoDrop Technologies, Rockland, DE, USA). Samples with total RNA concentration < 50 ng/μL were excluded from analysis because 200 ng of input RNA in a 5 μL volume was needed for hybridization with 20 μL of probe set master mix.

### nCounter expression assay (NanoString®)

The NanoString nCounter Analysis System (NanoString Technologies, Seattle, WA, USA) was used to measure gene expression. This system measures the relative abundance of each mRNA transcript via a multiplexed hybridization assay and digital readouts of fluorescent probes [29]. We used an nCounter CodeSet (NanoString Technologies) containing biotinylated capture probes for the EGFR, ERBB2, ERBB3, ERBB4, and ESR1 genes and five housekeeping genes and reporter probes attached to color barcode tags, according to the nCounterTM code-set design. These were hybridized in solution to 200 ng of total RNA for 18 h at 65 °C according to the manufacturer’s instructions.

Hybridized samples were loaded into the nCounter Prep Station for post-hybridization processing. Hybridized samples were purified and immobilized on the deck of the Prep Station in a sample cartridge for data collection, and target mRNA was quantified in each sample using the nCounter^TM^ Digital Analyzer. Quantified expression data were analyzed using NanoString nSolver Analysis Software.

After performing image quality control using a predefined cutoff value, we excluded outlier samples using a normalization factor based on the sum of positive control counts greater than threefold. The counts of the probes were then normalized using the geometric mean of the five housekeeping genes and log_2_ transformed for further analysis.

### Statistical analysis

Differences in clinicopathologic characteristics were analyzed using Student’s *t*-test for continuous variables and Pearson chi-square test for categorical variables. Distant relapse-free survival (DRFS) was defined as the elapsed time from the date of curative surgery to the detection of distant relapse of breast cancer. DRFS was analyzed by the Kaplan-Meier (KM) method. Univariate and multivariate analyses of DRFS were performed using Cox’s proportional hazards regression tests. To evaluate relationships among the expression levels of the five genes, we used Pearson correlation analysis. Finally, receiver operating characteristic (ROC) analysis was performed to evaluate the prognostic value of the level of gene expression. ROC analysis was conducted using weighted variables that were significantly associated with prognosis in previous univariate and multivariate analysis. Weighting of variables was performed using the hazard ratio in multivariate analysis. Two-tailed *p* values <0.05 were considered statistically significant, and IBM SPSS Statistics 21 for Windows (IBM Corp., Armonk, NY, USA) was used to analyze all data.

### Remark guidelines

**Fig. 1:**
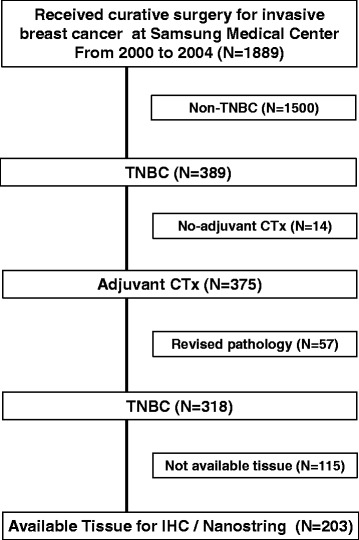
Patient cohort (*N* = 203)

In reporting our study, we have adhered to the guidelines of an important methodological paper from 2005 titled “Reporting recommendations for tumor marker prognostic studies (REMARK guidelines) [30].” To decrease any potential bias arising from a review of the medical records, we included “Patient Cohort” analysis to fulfill these criteria (Fig. 1).

## Results

### Baseline characteristics of patients

A total of 203 patients with TNBC were included in the training cohort (Fig. 1). In addition, 84 patients with TNBC were retrospectively included in a validation cohort. This validation cohort was composed of patients diagnosed with invasive breast cancer who received curative surgery from 2005 to 2010, regardless of chemotherapy status.

The baseline characteristics of patients in the training and validation cohorts are presented in Table 1. In the training cohort, the median age at diagnosis of breast cancer was 46.4 years (range, 23.5-74.1). Most patients were diagnosed with invasive ductal carcinoma (IDC) (88.7 %) and stage I-II disease (27.1 % as stage I and 62.6 % as stage II).

**Table 1:** Baseline characteristics of patient cohorts

	Training *N* = 203 (%)	Validation *N* = 84 (%)	*p*-value
Age (median)	46.4 ± 10.2	46.1 ± 11.0	0.308
Range	23.5-74.1	22.4 – 74.0	
< 40 years	48 (23.6)	28 (33.3)	
≥ 40 years	155 (76.4)	56 (66.7)	
Histology			0.654
IDC^a^	180 (88.7)	76 (90.5)	
Others	23 (11.3)	8 (9.5)	
Stage			<0.001
I	55 (27.1)	15 (17.9)	
IIA	94 (46.3)	18 (21.4)	
IIB	33 (16.3)	9 (11.9)	
IIIA	13 (6.4)	26 (31.0)	
IIIB	0 (0)	1 (1.2)	
IIIC	8 (3.9)	14 (16.7)	
Unknown	0 (0)	1 (1.2)	
Nuclear grade			0.191
1	2 (1.0)	0 (0)	
2	47 (23.2)	18 (21.4)	
3	145 (71.4)	57 (67.9)	
Unknown	9 (4.4)	9 (10.7)	
Histologic grade			0.411
1	3 (1.5)	1 (1.2)	
2	45 (22.2)	20 (23.8)	
3	144 (70.9)	54 (64.3)	
Unknown	11 (5.4)	9 (10.7)	
RNA expression (log2 scale, median)			
EGFR	7.0 ± 1.1	7.6 ± 1.2	0.456
ERBB2	9.0 ± 1.0	9.4 ± 1.6	0.439
ERBB3	7.2 ± 1.0	7.7 ± 1.0	0.503
ERBB4	1.3 ± 1.4	2.3 ± 2.2	0.302
ESR1	4.4 ± 0.1	4.5 ± 2.0	0.425
Chemotherapy			NA
Adjuvant	203 (100)	60 (71.4)	
Neoadjuvant	0 (0)	24 (28.6)	
Unknown	0 (0)	0 (0)	
Regimen			NA
CMF^1^	86 (42.4)	10 (11.9)	
FAC^2^	58 (28.6)	16 (19.0)	
AC^3^	17 (8.4)	5 (6.0)	
AC –T(H)^4^	41 (20.2)	46 (54.8)	
Hormone	0 (0)	0 (0)	
Unknown	1 (0.5)	7 (8.3)	
Adjuvant RTx^5^			0.161
Yes	130 (64.0)	61 (72.6)	
No	73 (36.0)	23 (27.4)	

^a^Invasive ductal carcinoma, ^1^Cyclophosphamide/Methotrexate/Fluorouracil, ^2^Fluorouracil/Adriamycin/Cyclophosphamide, ^3^Adriamycin/Cyclophosphamide, ^4^Taxane (Herceptin), ^5^Radiotherapy

The baseline characteristics were similar in training and validation cohorts. However, the patients in the validation cohort had a higher stage of breast cancer than those in the training cohort (*p* < 0.001).

### Gene expression profile of HER family genes and the ESR1 gene

**Fig. 2:**
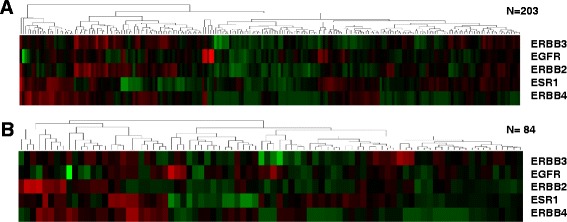
Heatmap for HER family and *ESR1* gene expression. **a** Training set (*N* = 203); **b** Validation set (*N* = 84)

The expression profiles of HER family genes and the ESR1 gene are presented in Fig. 2. Even within TNBC, each tumor sample had a distinct expression profile. However, the results of the nCounter expression assay showed that the level of EGFR, ERBB2, ERBB3, ERBB4, and ESR1 expression was similar in the training and validation cohorts (Table 1).

We also found that TNBC in the training and validation cohorts had lower expression of ERBB2, ERBB3, ERBB4, and ESR1 than non-TNBC subtypes with statistical significance (Additional file 1: Table S1 and Additional file 2: Figure S1). However, EGFR expression did not differ between TNBC and non-TNBC subtypes (*p* = 0.825). For further survival analysis, we set the median expression score of the five genes as the cut-off value to divide patients into low expression and high expression groups.

The association among the expression levels of the five genes was analyzed by Pearson correlation analysis. We found that ERBB2 expression in TNBC was positively correlated with ERBB3 expression (Pearson r = 0.651, *p* < 0.001), as well as with ERBB4 and ESR1 expression (Pearson r = 0.414, *p* < 0.001) (Additional file 2: Figure S2 and Additional file 1: Table S2).

### Effect of ERBB4 expression on distant relapse-free survival: univariate and multivariate analysis

**Fig. 3:**
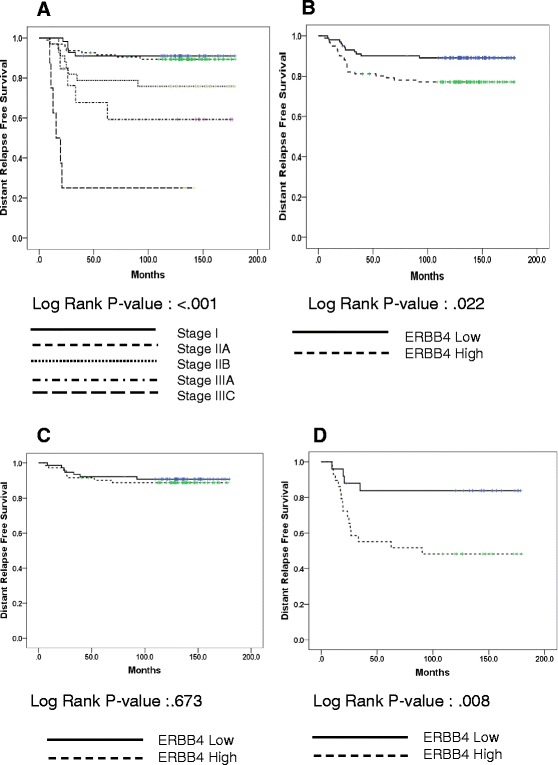
Survival analysis in the training set (*N* = 203). **a** Kaplan-Meier survival curve for stage at diagnosis. **b** Kaplan-Meier survival curve for level of ERBB4 expression. **c** Kaplan-Meier survival curve for level of ERBB4 expression in stage I/IIA (*N* = 149). **d** Kaplan-Meier survival curve for level of ERBB4 expression in stage IIB/IIIA/IIIC (*N* = 54)

Univariate analysis was conducted to investigate the effect of baseline characteristics on distant relapse-free survival (Table 2). The 5-year DRFS rate in patients with stage I and IIA disease was 90.9 % and 91.5 %, respectively, in contrast to patients with stage IIB, IIIA, and IIIC disease, who had 5Y DRFS of 78.8 %, 67.7 %, and 25.0 %, respectively (*p* < 0.001 by KM survival analysis) (Table 2 and Fig. 3a). Of gene expressions, patients with high-ERBB4 TNBC had 5Y DRFS of 80.2 %, compared with 90.1 % for those with low ERBB4 expression (*p* = 0.022) (Table 2 and Fig. 3b). We also found that patients who received taxane-containing chemotherapy had poor prognosis. However, we removed the variable of chemotherapy regimen for further statistical analysis due to the chemotherapy regimen was highly related to the stage of disease (*p* < 0.001 by chi-square test) (Additional file 1: Table S3).

**Table 2:** Impact of baseline characteristics on patient prognosis in the training cohort (*N* = 203)

	Training *N* = 203 (%)	5-year disease relapse-free survival (%)	*p*-value (Log-rank)
Age (median)	46.4 ± 10.2		
Range	23.5-74.1		0.633
< 40 years	48 (23.6)	81.2	
≥ 40 years	155 (76.4)	86.4	
Histology			0.507
IDC^a^	180 (88.7)	85.5	
Others	23 (11.3)	82.9	
Stage			<0.001
I	55 (27.1)	90.9	
IIA	94 (46.3)	91.5	
IIB	33 (16.3)	78.8	
IIIA	13 (6.4)	67.7	
IIIB	0 (0)		
IIIC	8 (3.9)	25.0	
Unknown	0 (0)		
Nuclear grade			0.258
1	2 (1.0)	50.0	
2	47 (23.2)	82.8	
3	145 (71.4)	86.9	
Unknown	9 (4.4)	77.8	
Histologic grade			0.704
1	3 (1.5)	100.0	
2	45 (22.2)	84.4	
3	144 (70.9)	86.0	
Unknown	11 (5.4)	72.7	
EGFR (median: 7.0)			0.084
Low	104 (51.2)	90.3	
High	99 (48.8)	79.8	
ERBB2 (median: 9.0)			0.402
Low	102 (50.2)	88.2	
High	101 (49.8)	82.1	
ERBB3 (median: 7.2)			0.106
Low	102 (50.2)	90.2	
High	101 (49.8)	80.1	
ERBB4 (median: 1.3)			0.022
Low	102 (50.2)	90.1	
High	101 (49.8)	80.2	
ESR1 (median: 4.4)			0.689
Low	102 (50.2)	84.2	
High	101 (49.8)	86.1	
Adjuvant chemotherapy			0.001
CMF^1^	86 (42.4)	90.7	
FAC^2^	58 (28.6)	86.0	
AC^3^	17 (8.4)	100.0	
AC –T^4^	41 (20.2)	65.9	
Unknown	1 (0.5)	100.0	
Adjuvant RTx^5^			0.093
Yes	130 (64.0)	83.0	
No	73 (36.0)	89.0	

^a^Invasive ductal carcinoma, ^1^Cyclophosphamide /Methotrexate/Fluorouracil, ^2^Fluorouracil/Adriamycin/Cyclophosphamide, ^3^Adriamycin/Cyclophosphamide, ^4^Taxane, ^5^Radiotherapy

In multivariate analysis using stage and ERBB4 gene expression, both variables remained statistically significant prognostic factors for DRFS: hazard ratio (HR) of DRFS 1.37 (95 % confidence interval [CI] 0.47-4.01 for stage IIA; HR 3.28 (95 % CI 1.07-10.04) for stage IIB; HR 4.81, (95 % CI 1.39-16.65), for stage IIIA, and HR 35.12, (95 % CI 9.62-128.27) for IIIC; HR 3.12 (95 % CI 1.42-6.87) for high expression of ERBB4 (Table 3A).

**Table 3:** Effect of mRNA expression levels of ERBB4 and stage on DRFS (multivariate analysis, Cox-regression)

Clinical variables	HR	95% CI	*p*-value
(A) All stages			
Stage			<0.001
I	1.0	NA	
IIA	1.37	0.47 – 4.01	
IIB	3.28	1.07 – 10.04	
IIIA	4.81	1.39 – 16.65	
IIIC	35.12	9.62 – 128.27	
ERBB4 (median: 1.3)			0.005
Low	1.0	NA	
High	3.12	1.42 – 6.87	
(B) Early stage (*N* = 149)			
Stage			0.669
I	1.0	NA	
IIA	1.37	0.43 – 3.77	
ERBB4 (median: 1.3)			0.728
Low	1.0	NA	
High	1.21	0.41 – 3.55	
(C) Advanced stage (*N* = 54)			
Stage			<0.001
IIB	1.0	NA	
IIIA	1.12	0.33 – 3.83	
IIIC	35.71	6.73 – 189.65	
ERBB4 (median: 1.3)			0.004
Low	1.0	NA	
High	7.79	1.96 – 31.01	

Furthermore, we analyzed the relationship between ERBB4 expression and DRFS according to stage. Because previous analysis showed that patients with stages I or IIA had similar survival rates whereas those with more advanced stage had poor prognosis, we divided patients into an early-stage group (stage I/IIA) and an advanced-stage group (stage IIB/IIIA/IIIC). In early-stage breast cancer, ERBB4 expression did not affect patient survival (5Y DRFS, low vs. high: 90.9 % vs. 90.2 %, *p* = 0.672) (Fig. 3c and Table 3B). However, for patients diagnosed at advanced stage, those with high ERBB4 expression had significantly poorer survival outcomes than those with low ERBB4 expression (5Y DRFS, low vs. high: 83.8 % vs. 55.2 %, *p* = 0.008) (Fig. 3d and Table 3C).

**Fig. 4:**
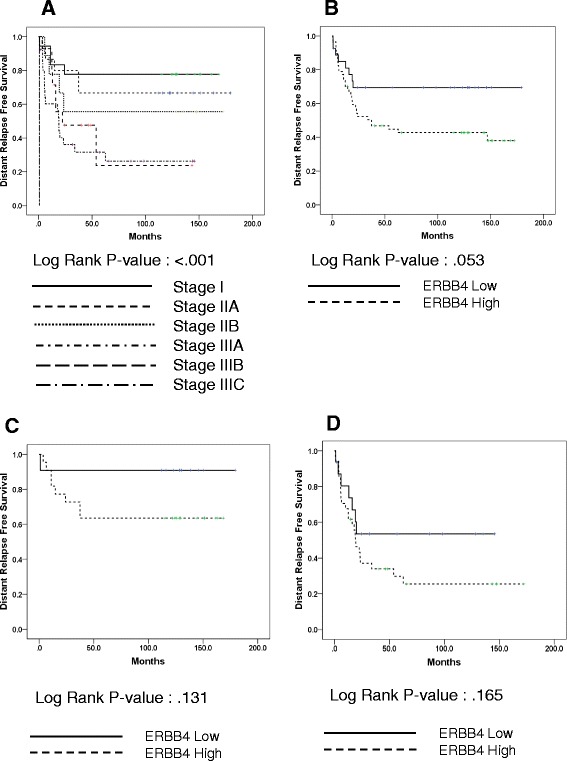
Survival analysis in the validation set (*N* = 84). **a** Kaplan-Meier survival curve for stage at diagnosis. **b** Kaplan-Meier survival curve for level of ERBB4 expression. **c** Kaplan-Meier survival curve for level of ERBB4 expression in stage I/IIA (*N* = 33). **d** Kaplan-Meier survival curve for level of ERBB4 expression in stage IIB/IIIA/IIIC (*N* = 51)

We performed survival analysis on the validation set. Patients in the validation set with high ERBB4-expressing breast cancer (cut-off value (log2 scale) = 1.3, the same as that used for the training set) had worse DRFS rates than those with low expression (5Y DRFS, low vs. high: 69.4 % vs. 44.7 %, *p* = 0.053) (Fig. 4b). We also observed this trend after dividing the patients into subgroups of early stage and advanced stage, although it lacked statistical significance (Fig. 4c and 4d). In addition, we analyzed relationship between ERBB4 expression and patients’ prognosis in the control group. In contrast of TNBC, this analysis showed that breast cancer with high ERBB4 expression had better prognosis than that with low ERBB4 expression (*p* = 0.003), as like as previous research presenting that ERBB4 overexpression was good prognostic indicator of ER positive and/or HER2 positive breast cancer.

### Effect of interaction between ERBB4 and ESR1 gene expression on distant relapse-free survival in TNBC

To elucidate the interaction between ERBB4 and ESR1 gene expression, we analyzed the impact of ERBB4 and ESR1 expression on DRFS in patients with TNBC.

**Fig. 5:**
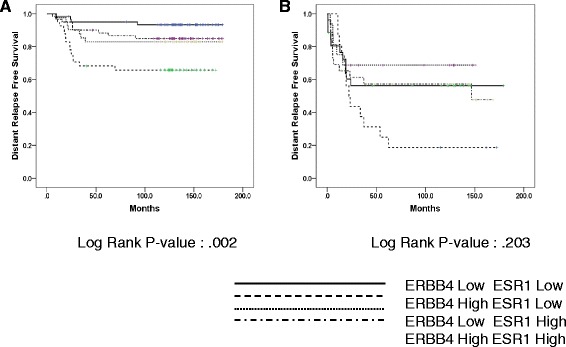
Survival analysis according to the level of ESR1 and ERBB4 expression. **a** Training set (*N* = 203). **b** Validation set (*N* = 84)

We found that the group of high ERBB4 and low ESR1 expression had the worst DRFS duration whereas patients with low ERBB4 and low ESR1 expression had the longest DRFS (p = 0.002) (Fig. 5a). This trend remained but without statistical significance in the validation set (Fig. 5b) and in subgroup analyses according to early-stage and advanced-stage breast cancer (Additional file 2: Figure S3A and 3B).

### Prognostic value of the level of ERBB4 expression in TNBC

**Fig. 6:**
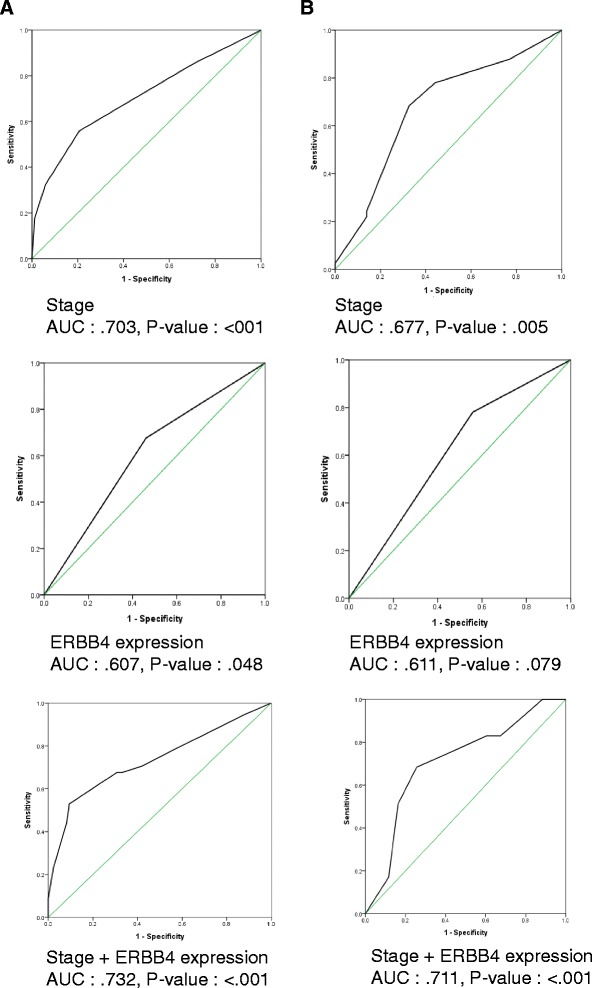
ROC analysis of predictive accuracy of stage and ERBB4 expression for DRFS. **a** Training set (*N* = 203). **b** Validation set (*N* = 84)

ROC analysis was performed to evaluate the prognostic value of ERBB4 expression level. According to multivariate analysis, addition of ERBB4 expression level to TNM stage enabled prediction of DRFS in both the training and the validation set (Fig. 6). The results of ROC analysis revealed that the value of ERBB4 expression strengthened the predictive efficacy of TNM stage at diagnosis in both the training and the validation set. In the training set, the AUC of the expression level of ERBB4 with TNM stage was 0.732 (*p* < 0.001), which is superior to the individual predictive values of ERBB4 expression and TNM stage (TNM stage: AUC 0.703, *p* < 0.001; ERBB4 expression: AUC 0.607, *p* = 0.048) (Fig. 6a). We confirmed that this trend was also evident in the validation set (TNM stage: AUC 0.677, *p* = 0.005; ERBB4 expression: AUC 0.611, *p* = 0.079; ERBB4 expression + TNM stage: AUC 0.711, *p* < 0.001; Fig. 6b).

## Discussion

In this study, we demonstrated the role of HER family genes in TNBC and we suggested that the level of ERBB4 expression had potential prognostic value in TNBC.

Many studies on HER family gene expression in breast cancer have previously been performed. Although most of these studies involved breast cancer with EGFR or ERBB2 overexpression, some researchers have conducted studies on the other HER family genes, including ERBB4. These studies revealed that ERBB4 overexpression in breast cancer is correlated with hormone receptor positivity [13, 20] and/or ERBB3 overexpression [31]. In addition, ERBB4 overexpression was reported to be associated with favorable prognosis in breast cancer patients [14], especially in cases of ER-positive [18, 31] and/or ERBB2-amplified breast cancer [20, 32]. In contrast, Bieche et al. showed that ERBB4-expression of breast cancer more than that of normal breast tissue had extremely poor prognosis compared with ERBB4-underexpressing breast cancer [17]. This study also included subgroup analysis that identified ERBB4 overexpression as a marker for poor prognosis in ER-negative breast cancer.

In our study, HER family gene expression was measured by nCounter expression assay. Because we used formalin fixed paraffin embedded (FFPE) tissues which collected 10 years ago, we needed a more sensitive technology for detection of gene expression to overcome the weakness of IHC using old FFPE samples. Moreover, in contrast to EGFR and ERBB2, immunohistochemistry of ERBB3 and ERBB4 is not well established. Accordingly, we chose to use the nCounter expression assay as the method to measure gene expression rather than IHC. Therefore, although this study was conducted on triple negative breast cancer, we were able to detect RNA expression of ERBB2 and ESR1, and found a complex expression pattern among HER family members and the ESR1 gene in TNBC. Moreover, our research showed that ERBB4 expression could serve as a potential prognostic factor when combined with pathologic stage in TNBC, and confirmed this result in the validation set. In a subgroup analysis, we found that the significance of ERBB4 expression was more prominent in advanced-stage TNBC.

Many tyrosine kinase inhibitors and monoclonal antibodies against EGFR and ERBB2 RTK have successfully been used as cancer drugs, for example cetuximab [33] and panitumumab [34] for EGFR overexpression, and afatinib [35] and dacomitinib as pan-HER inhibitors. In the clinic, trastuzumab [8], pertuzumab [10], lapatinib [11], and TDM-1[36] have been used in anti-HER2 therapy for ERBB2-overexpressing breast cancer. However, the biologic role of ERBB4 and its potential as a target for cancer drugs has not been clearly identified. Some studies have reported that ERBB4 regulated cell differentiation and cell survival via the Mek/Erk pathway [37, 38] and lapatinib inhibited the interaction of ERBB4 with EGFR and ERBB2 [39]. Therefore, lapatinib or a MEK inhibitor might be an effective therapeutic option in TNBC with high ERBB4 expression.

This study is the first study to demonstrate the impact of ERBB4 expression on patient prognosis in TNBC. Our study revealed that high expression of ERBB4 is an independent prognostic factor in TNBC. Because this study was performed in two independent groups, a training set and a validation set, the results of this study are of high validity and reliability.

Our study did not include analysis of patient overall survival. Because overall survival is influenced by patient’s characteristics like age, comorbidities as well as breast cancer characteristics, DRFS might be more reliable indicator for breast cancer-specific survival rather than overall survival.

In conclusion, our research suggests that ERBB4 expression is a valuable prognostic marker and may be useful to predict response to therapy for triple negative breast cancer. Moreover, we expect that further clinical trials on RTKs would benefit patients suffering from refractory triple negative breast cancer with high ERBB4 expression.

## Conclusions

Our research suggests that ERBB4 expression is a useful prognostic marker and may be useful to predict response to therapy for triple negative breast cancer.

## Additional files

Additional file 1: Table S1. Baseline characteristics of patients’ cohort. **Table S2.** The relationship among expression levels of ERBB family RTKs in TNBC. **Table S3. **elationships between stage of disease and chemotherapeutic agent. **Table S4.** Univariate analysis of baseline characteristics affecting ERBB4 expression (N=203). (DOCX 29 kb) Additional file 2: Figure S1. Box plots for the expression level of HER family genes and *ESR1* gene. (A) Training set (*N* = 203). (B) Validation set (*N* = 84). (C) Group consisting of non-triple negative breast cancer (*N* = 52). **Figure S2.** Relationship among expression of the ERBB family genes and *ESR1* gene in TNBC. **Figure S3.** Survival analysis according to the expression level of ESR1 and ERBB4 expression in the training set. (A) Kaplan-Meier survival curve for stage I/IIA (*N* = 149). (B) Kaplan-Meier survival curve for stage IIB/IIIA/IIIC (*N* = 54). (PPTX 266 kb)

## Abbreviations

HER: Human epidermal growth factor receptor
TNBC: Triple negative breast cancer
ESR1: estrogen receptor 1
EGFR: Epidermal growth factor receptor
ERBB2: Erb-B2 Receptor Tyrosine Kinase 2
ERBB3: Erb-B2 Receptor Tyrosine Kinase 3
ERBB4: Erb-B2 Receptor Tyrosine Kinase 4
ER: Estrogen receptor
PgR: Progesterone receptor
FFPE: Formalin-fixed paraffin-embedded
DRFS: Distant relapse-free survival
KM: Kaplan-Meier
ROC: Receiver operating characteristic
IDC: Invasive ductal carcinoma
HR: Hazard ratio
