# Supplementary material for: Prognostic value of ERBB4 expression in patients with triple negative breast cancer
Source: BMC Cancer. 2016 Feb 22;16:138. doi: 10.1186/s12885-016-2195-3 (PMC4763414; doi:10.1186/s12885-016-2195-3)
Supplement: Additional file 1: Table S1. — Baseline characteristics of patients’ cohort. Table S2. The relationship among expression levels of ERBB family RTKs in TNBC. Table S3. elationships between stage of disease and chemotherapeutic agent. Table S4. Univariate analysis of baseline characteristics affecting ERBB4 expression (N=203). (DOCX 29 kb) [file 12885_2016_2195_MOESM1_ESM.docx]

**Supplementary Table1. Baseline characteristics of patients’ cohort**

|  | **Training**  **N=203(%)** | **Validation**  **N=84(%)** | **Control**  **N=52(%)** | **P-value** |
| --- | --- | --- | --- | --- |
| Age(Median) | 46.4±10.2 | 46.1±11.0 | 44.3 ± 8.3 | .199 |
| Range | 23.5-74.1 | 22.4 – 74.0 | 27.0 – 60.0 |  |
| <40 YO* | 48 (23.6) | 28 (33.3) | 16 (30.8) |  |
| ≥40 YO | 155 (76.4) | 56 (66.7) | 36 (69.2) |  |
| Histology |  |  |  | .267 |
| IDC† | 180 (88.7) | 76 (90.5) | 50 (96.2) |  |
| Others | 23 (11.3) | 8 (9.5) | 2 (3.8) |  |
| Subtype |  |  |  | NA |
| ER+/HER2- | 0 (0) | 0 (0) | 28 (53.8) |  |
| ER+/HER2+ | 0 (0) | 0 (0) | 10 (19.2) |  |
| ER- /HER2+ | 0 (0) | 0 (0) | 14 (26.9) |  |
| TNBC‡ | 203 (100) | 84 (100) | 0 (0) |  |
| Stage |  |  |  | <.001 |
| I | 55 (27.1) | 15 (17.9) | 0 (0) |  |
| IIA | 94 (46.3) | 18 (21.4) | 0 (0) |  |
| IIB | 33 (16.3) | 9 (11.9) | 1 (1.9) |  |
| IIIA | 13 (6.4) | 26 (31.0) | 39 (75.0) |  |
| IIIB | 0 (0) | 1 (1.2) | 2 (3.8) |  |
| IIIC | 8 (3.9) | 14 (16.7) | 10 (19.2) |  |
| Unknown | 0 (0) | 1 (1.2) | 0 (0) |  |
| Nuclear grade |  |  |  | <.001 |
| 1 | 2 (1.0) | 0 (0) | 5 (9.6) |  |
| 2 | 47 (23.2) | 18 (21.4) | 11 (21.2) |  |
| 3 | 145 (71.4) | 57 (67.9) | 28 (53.8) |  |
| Unknown | 9 (4.4) | 9 (10.7) | 8 (15.4) |  |
| Histologic grade |  |  |  | <.001 |
| 1 | 3 (1.5) | 1 (1.2) | 6 (11.5) |  |
| 2 | 45 (22.2) | 20 (23.8) | 21 (40.4) |  |
| 3 | 144 (70.9) | 54 (64.3) | 11 (21.2) |  |
| Unknown | 11 (5.4) | 9 (10.7) | 14 (26.9) |  |
| RNA expression  (log2 scale, Median) |  |  |  |  |
| EGFR | 7.0 ± 1.1 | 7.6 ± 1.2 | 7.1 ± 8.2 | .825 |
| ERBB2 | 9.0 ± 1.0 | 9.4 ± 1.6 | 13.3 ± 15.4 | <.001 |
| ERBB3 | 7.2 ± 1.0 | 7.7 ± 1.0 | 8.6 ± 9.5 | <.001 |
| ERBB4. | 1.3 ± 1.4 | 2.3 ± 2.2 | 4.8 ± 8.8 | <.001 |
| ESR1 | 4.4 ± 0.1 | 4.5 ± 2.0 | 8.9 ± 12.5 | <.001 |
| Chemotherapy |  |  |  | NA |
| Adjuvant | 203 (100) | 60 (71.4) | 0 (0) |  |
| Neoadjuvant | 0 (0) | 24 (28.6) | 52 (100) |  |
| Unknown | 0 (0) | 0 (0) | 0 (0) |  |
| Regimen |  |  |  | NA |
| CMF^1^ | 86 (42.4) | 10 (11.9) | 0 (0) |  |
| FAC^2^ | 58 (28.6) | 16 (19.0) | 0 (0) |  |
| AC^3^ | 17 (8.4) | 5 (6.0) | 1 (1.9) |  |
| AC –T(H)^4^ | 41 (20.2) | 46 (54.8) | 51 (98.1) |  |
| Hormone | 0 (0) | 0 (0) | 36 (69.2) |  |
| Unknown | 1 (0.5) | 7 (8.3) | 0(0) |  |
| Adjuvant RTx^5^ |  |  |  | <.001 |
| Yes | 130 (64.0) | 61 (72.6) | 52 (100) |  |
| No | 73 (36.0) | 23 (27.4) | 0 (0) |  |

^*^Years old, ^†^Invasive ductal carcinoma, ^‡^Triple-negative breast cancer, ^1^Cyclophosphamide/Methotrexate/Fluorouracil, ^2^Fluorouracil/Adriamycin/Cyclophosphamide, ^3^Adriamycin/Cyclophosphamide, ^4^Taxane (Herceptin), ^5^Radiotherapy

**Supplementary Table 2. The relationship among expression levels of ERBB family RTKs in TNBC**

| **Clinical variables** | **EGFR** | **ERBB2** | **ERBB3** | **ERBB4** | **ESR1** |
| --- | --- | --- | --- | --- | --- |
| EGFR |  |  |  |  |  |
| Pearson R | 1 | -0.056 | -0.048 | 0.022 | -0.22 |
| *p*-value |  | 0.430 | 0.495 | 0.761 | 0.774 |
| ERBB2 |  |  |  |  |  |
| Pearson R | -0.56 | 1 | 0.651 | 0.123 | 0.232 |
| *p*-value | 0.430 |  | < 0.001 | 0.081 | 0.001 |
| ERBB3 |  |  |  |  |  |
| Pearson R | -0.048 | 0.651 | 1 | 0.162 | 0.109 |
| *p*-value | 0.495 | < 0.001 |  | 0.021 | 0.120 |
| ERBB4 |  |  |  |  |  |
| Pearson R | 0.022 | 0.123 | 0.162 | 1 | 0.414 |
| *p*-value | 0.761 | 0.081 | 0.021 |  | < 0.001 |
| ESR1 |  |  |  |  |  |
| Pearson R | -0.020 | 0.232 | 0.109 | 0.414 | 1 |
| *p*-value | 0.774 | 0.001 | 0.120 | < 0.001 |  |

**Supplementary Table 3. Relationships between stage of disease and chemotherapeutic agent**

| **Stage** | **CMF^1^(%)** | **FAC^2^ (%)** | **AC^3^ (%)** | **AC-T^4^ (%)** | **Unknown (%)** | ***p*-value** |
| --- | --- | --- | --- | --- | --- | --- |
| I | 34(61.8) | 16(29.1) | 5(9.1) | 0(0) | 0(0) | < 0.001 |
| IIA | 46 (48.9) | 32(34.0) | 7(7.4) | 9(9.6) | 0(0) |  |
| IIB | 6(18.2) | 5(15.2) | 5(15.2) | 16(48.5) | 1(3.0) |  |
| IIIA | 0(0) | 4(30.8) | 0(0) | 9(69.2) | 0(0) |  |
| IIIC | 0(0) | 1(12.5) | 0(0) | 7(87.5) | 0(0) |  |

^1^Cyclophosphamide /Methotrexate/Fluorouracil, ^2^Fluorouracil/Adriamycin/Cyclophosphamide, ^3^Adriamycin/Cyclophosphamide, ^4^Taxane

**Supplementary Table4. Univariate analysis of baseline characteristics affecting ERBB4 expression (N=203)**

|  | **Low ERBB4 (N=102)** | **High ERBB4(N=101)** | **P-value** |
| --- | --- | --- | --- |
| Age(Median) | 46.4±10.2 |  |  |
| Range | 23.5-74.1 |  | .969 |
| <40 YO^*^ | 24 (23.5) | 24 (23.8) |  |
| ≥40 YO | 78 (76.5) | 77 (76.2) |  |
| Histology |  |  | .523 |
| IDC^†^ | 89 (87.3) | 91 (90.1) |  |
| Others | 13 (12.7) | 10 (9.9) |  |
| Stage |  |  | .301 |
| I | 24 (23.5) | 31 (30.7) |  |
| IIA | 53 (52.0) | 41 (40.6) |  |
| IIB | 16 (15.7) | 17 (16.8) |  |
| IIIA | 4 (3.9) | 9 (8.9) |  |
| IIIB | 0 (0) | 0 (0) |  |
| IIIC | 5 (4.9) | 3 (3.0) |  |
| Unknown | 0 (0) | 0 (0) |  |
| Nuclear grade |  |  | .263 |
| 1 | 0 (0) | 2 (2.0) |  |
| 2 | 20 (19.6) | 27 (26.7) |  |
| 3 | 78 (76.5) | 67 (66.3) |  |
| Unknown | 4 (3.9) | 5 (5.0) |  |
| Histologic grade |  |  | .116 |
| 1 | 1 (1.0) | 2 (2.0) |  |
| 2 | 16 (15.7) | 29 (28.7) |  |
| 3 | 78 (76.5) | 66 (65.3) |  |
| Unknown | 7 (6.9) | 4 (4.0) |  |
| Adjuvant CTx. |  |  | .318 |
| CMF^1^ | 43 (42.2) | 43 (42.6) |  |
| FAC^2^ | 31 (30.4) | 27 (26.7) |  |
| AC^3^ | 11 (10.8) | 6 (5.9) |  |
| AC –T^4^ | 16 (15.7) | 25 (24.8) |  |
| Unknown | 1 (1.0) | 0 (0) |  |
| Adjuvant RTx^5^ |  |  | .497 |
| Yes | 63 (61.8) | 67 (66.3) |  |
| No | 39 (38.2) | 34 (33.7) |  |

^*^Year Old, ^†^Invasive ductal carcinoma, ^1^Cyclophospamide /Metotraxate/Fluorouracil, ^2^Fluorouracil/Adriamycin/Cyclophosphamide, ^3^Adriamycin/Cyclophosphamide, ^4^Taxane, ^5^Radiotherapy
