# Supplementary material for: Prognostic value of ERBB4 expression in patients with triple negative breast cancer
Source: BMC Cancer. 2016 Feb 22;16:138. doi: 10.1186/s12885-016-2195-3 (PMC4763414; doi:10.1186/s12885-016-2195-3)
Supplement: Additional file 2: Figure S1. — Box plots for the expression level of HER family genes and ESR1 gene. (A) Training set (N = 203). (B) Validation set (N = 84). (C) Group consisting of non-triple negative breast cancer (N = 52). Figure S2. Relationship among expression of the ERBB family genes and ESR1 gene in TNBC. Figure S3. Survival analysis according to the expression level of ESR1 and ERBB4 expression in the training set. (A) Kaplan-Meier survival curve for stage I/IIA (N = 149). (B) Kaplan-Meier survival curve for stage IIB/IIIA/IIIC (N = 54). (PPTX 266 kb) [file 12885_2016_2195_MOESM2_ESM.pptx]

## Slide 1
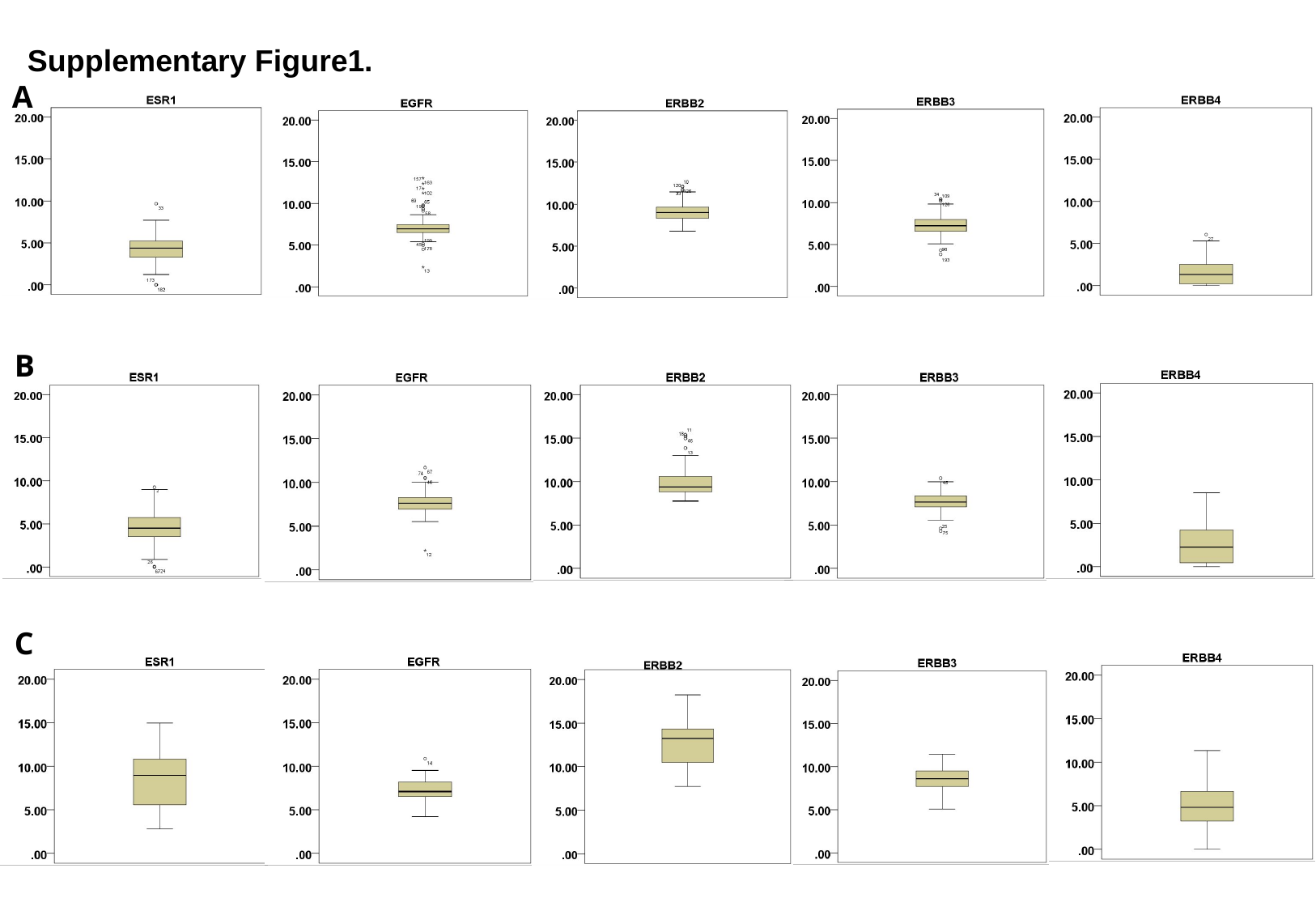

Supplementary Figure1.
A
B
C

## Slide 2
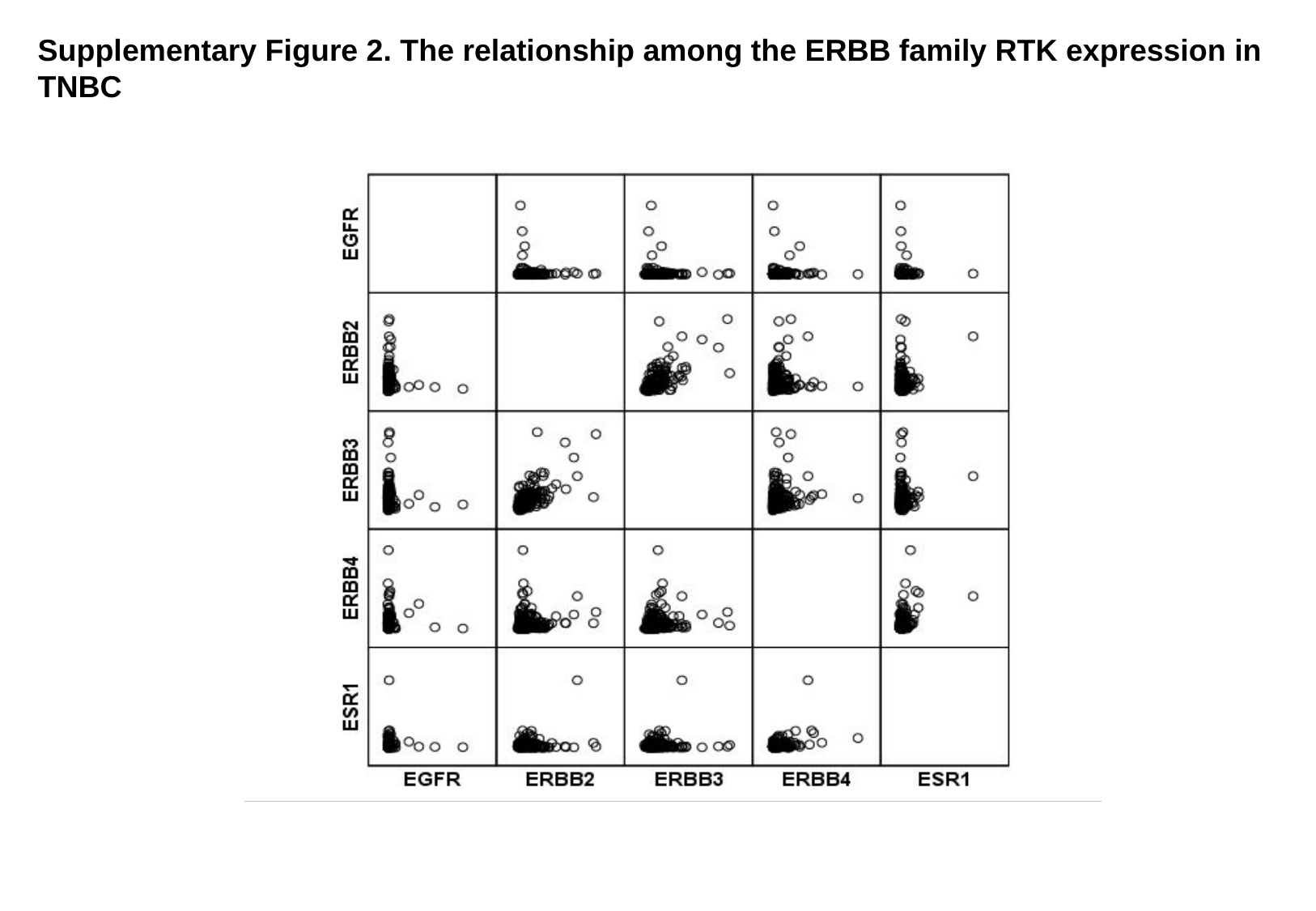

Supplementary Figure 2. The relationship among the ERBB family RTK expression in TNBC

## Slide 3
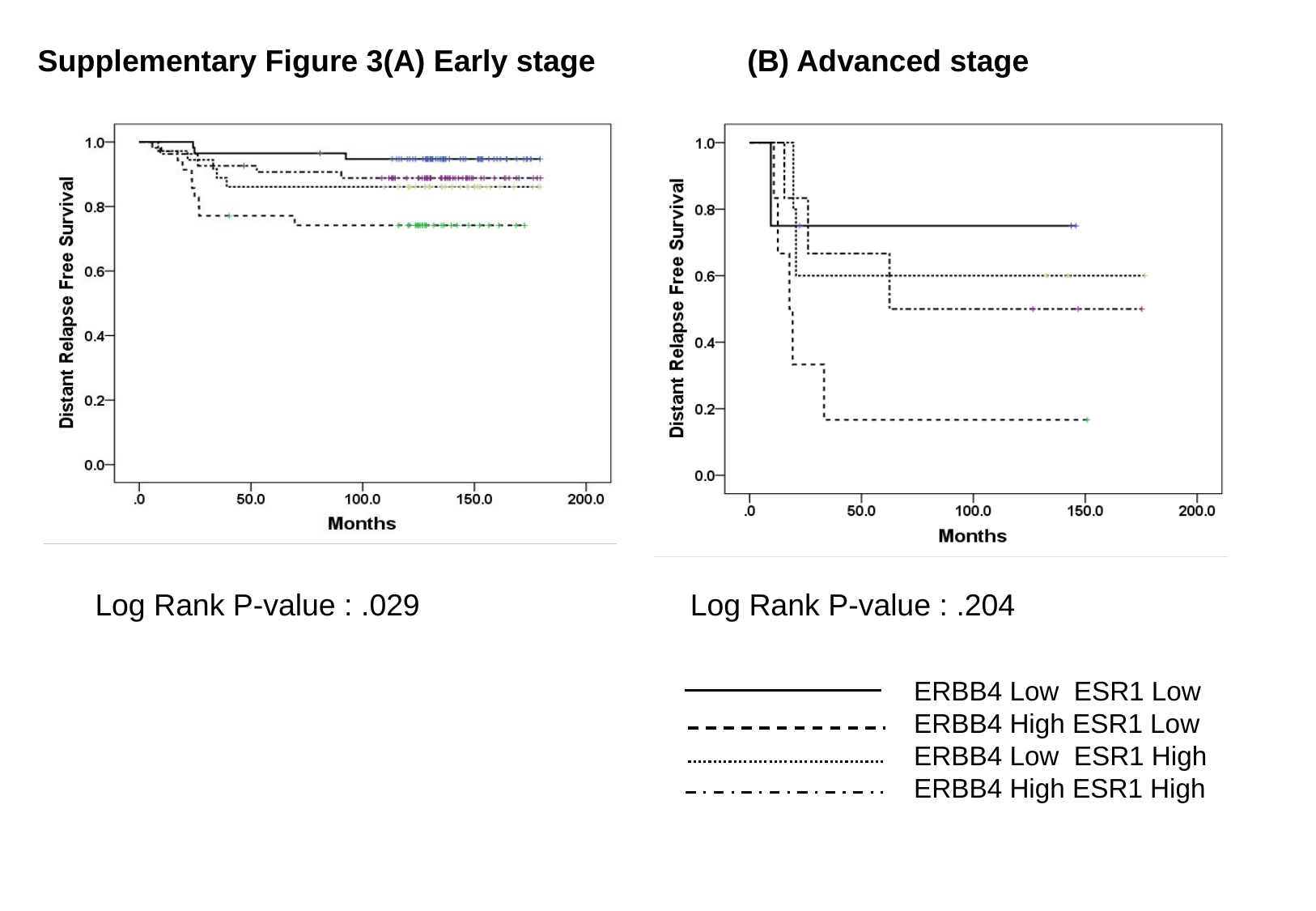

Supplementary Figure 3(A) Early stage
(B) Advanced stage
Log Rank P-value : .029
Log Rank P-value : .204
ERBB4 Low ESR1 Low
ERBB4 High ESR1 Low
ERBB4 Low ESR1 High
ERBB4 High ESR1 High
